# *Momordica charantia* extracts ameliorate insulin resistance by regulating the expression of SOCS-3 and JNK in type 2 diabetes mellitus rats

**DOI:** 10.1080/13880209.2017.1396350

**Published:** 2017-11-07

**Authors:** Chunyu Ma, Hongyu Yu, Ying Xiao, Huijiao Wang

**Affiliations:** a Department of Laboratory Diagnosis, Jinzhou Medical University, Jinzhou, PR China;; b Department of Basic Nursing, Jinzhou Medical University, Jinzhou, PR China

**Keywords:** Suppressor of cytokine signaling-3, c-Jun N-terminal kinase, liver glycogen, TNF-α, IL-6

## Abstract

**Context:***Momordica charantia* L. (Cucurbitaceae) has long been widely used as a traditional remedy for diabetes mellitus in some countries. However, detailed antidiabetic mechanisms are largely unknown.

**Objectives:** This study clarified the ameliorating effects of *M. charantia* ethanol extracts (MCE) on the insulin resistance in type 2 diabetes mellitus (T2DM) rats.

**Materials and methods:** T2DM rat model was established by high-fat diet and streptozotocin (STZ) injection. Diabetic rats were randomized into five groups: the model control group (*n* = 8) (common diet), the high-fat diet metformin (50 mg/kg/d), and the three-dose MCE (100, 200, and 400 mg/kg/d) groups (*n* = 8 each). After 8  weeks, the fasting serum glucose, insulin, TNF-α, and IL-6 were measured, and the relevant factors of glucose and insulin were monitored by glycogen dyeing, RT-PCR, and western blot, respectively.

**Results:** The 8-week treatment of 400 mg/kg MCE significantly lowered body weight (330.1 versus 365.9 g), serum glucose (7.41 versus 16.63 mmol/L), insulin (12.06 versus 15.89 mIU/L), TNF-α (52.72 versus 81.83 ng/L), and IL-6 (104.81 versus 135.74 ng/L) in comparison with those of the diabetic control group (*p* < 0.05). It was the same for skeletal muscle glucose transporter 4 (GLUT-4) protein, and glycogen level, suppressor of cytokine signaling-3 (SOCS-3), c-Jun N-terminal kinase (JNK), and Akt expression at both protein and mRNA levels in liver (*p* < 0.05).

**Conclusions:** MCE can ameliorate insulin resistance in T2DM rats. This effect may be related to the regulation of mRNA and protein levels of SOCS-3 and JNK.

## Introduction

The incidence of diabetes mellitus has reached epidemic proportions worldwide and it will rise to more than 300 million people by 2025 (Adeghate et al. 2006). Among these patients with diabetes, 90–95% have type 2 diabetes mellitus (T2DM) (Chun et al. 2014). Therefore, the prevention and control of T2DM has become a major health care focus. Anti-diabetic treatments are grouped into diet, exercise, and drugs. Currently, the T2DM treatment drugs include insulin and anti-hyperglycaemic drugs, such as sulphonylurea derivatives, biguanides, and thiazolidinediones. Additionally, several herbal remedies provide a novel method to treat T2DM, which are characterized by fewer side effects and lasting moderate effect. *Momordica charantia* L. (Cucurbitaceae) has been used in the management of hyperglycaemia and early signs of diabetes since ancient times in some countries (Yin et al. 2008; Leung et al. 2009; Efird et al. 2014). It is effective and non-toxin for patients compared with other hypoglycaemic agents (Habicht et al. 2014) although, *M. charantia* extract (MCE) has hypolipidaemic (Chaturvedi et al. 2004), weight loss (Chen et al. 2003), and anti-inflammatory (Chao et al. 2014) activities.

Insulin resistance (IR) is the principal pathogenesis of T2DM as an independent risk factor. It occurs with the decreasing expression of hepatic glycogen and GLUT-4 in insulin-receptor-positive cells. Hepatic glycogen has the effect on the adjusting of blood glucose balance. GLUT-4 can transfer glucose from blood to tissue for utilization. However, IR resulted from abnormal insulin and downstream PI3K/Akt signal pathways, which can modulate glucose transport, glycogen synthesis, glycolysis, and protein synthesis (White 2002). Moreover, there are several inhibitory molecules for insulin-signalling pathway such as the protein tyrosine phosphatase 1B (PTP-1B), the suppressor of cytokine signaling-3 (SOCS-3), and c-Jun N-terminal kinase (JNK). However, such inflammatory signals as TNF-α and IL-6 can activate inhibitory molecules such as SOCS-3 and JNK to suppress insulin-signalling pathway and cause IR (Kwon and Pessin 2013). Insulin receptor’s distribution density reduces in adipocyte and the typical pro-inflammatory cytokines such as TNF-α and IL-6 are increased in the obese humans and rodents, suggesting that these factors could contribute to IR. These pro-inflammatory cytokines have the potential to exert negative effects on insulin sensitivity in an endocrine or paracrine manner. Therefore, T2DM has been proposed as a kind of inflammatory disease (Donath and Shoelson 2011; Lee and Lee 2014).

In this study, in order to clarify the hypoglycaemic effects and illuminate the ameliorating IR’s mechanism of MCE, fasting blood glucose and insulin levels were tested. The serum TNF-α and IL-6 levels were also measured. Furthermore, the hepatic glycogen and the glucose transporter 4 (GLUT-4) expression was observed, and the insulin signal-related factors such as Akt-2, PTP-1B, SOCS-3, and JNK’s mRNA and proteins were also analyzed.

## Materials and methods

### The extract method of *M. charantia* ethanol extracts (MCE)

The mature green *M. charantia* were purchased from Jinzhou Darunfa super market in September 2012. This plant was taxonomically identified by Prof. Dr. Lijing Geng (College of Food Science, Jinzhou Medical University) and a voucher specimen (No. MC-120925) was deposited at the Herbarium of the College of Food Science, Jinzhou Medical University. The *M. charantia* fruits were washed thoroughly, and the seeds were removed. The pulp was cut into small pieces, dried, and smashed to powder, and then extracted with 70% ethanol (the ratio of material to solvent is 1–9) by 6 h. The ethanol extract was concentrated under the rotary evaporation, dried to solid by putting into freezer dryer, and then smashed to powder. The actual yield of MCE (freeze-dried powder versus dried weight of fruits) is 0.56%. The total saponins were identified by the vanillin-perchlorate chromogenic method using ultraviolet spectrophotometer (UV-2550 type, Shimadu, Japan). Ginsenoside Rg1 (MUST-13072505, Chengdu MUST Biological Technology Corporation, Chengdu, China) was selected as a reference substance and the absorbance was determined at 560 nm. Saponins content was calculated as the regression equation (*y* = 0.0017*x* − 0.1745). The total saponins content in MCE was 41.3%.

### Experimental animals

All SPF-grade male SD rats (Liaoning Changsheng Corporation, Dalian, China, Animal license number: SCXK2010-0001) were purchased and housed in an air-conditioned room at 22 ± 3 °C with common diet and tap water *ad libitum*. All animal experiments were carried out in accordance with the National Research Council Guide for the Care and Use of Laboratory Animals.

### Induction of T2DM rats and animal grouping

After a 1-week acclimation period, the rats were divided randomly into two groups. The control (CON) group (*n* = 8) was fed with common diet, whereas the experimental group (*n* = 40) was fed the high-fat diet (the standard fodder:sugar:lard:yolk =60:17:20:3) for 8 weeks. After 8 weeks high-fat diet, the 25 mg/kg dose of streptozotocin (STZ, dissolved in 0.05 M citrate buffer at pH 4.5, prepared immediately before use, Sigma Corporation, St. Louis, MO) was injected through caudal vein in the experimental group rats as previously described (Spasov et al. 2007). One week after the STZ injection, rats were assessed by fasting blood glucose from the tail vein. The fasting blood glucose ≥11.1 mmol/L was considered as a rat model of T2DM. Diabetic rats were fed with common diet and then were randomized into five groups: the model control group (*n* = 8), the metformin (Shandong Oriental Furuida Corporation, Dezhou, China) group (*n* = 8), and the three-dose MCE group (*n* = 24). The MCE group was administrated by oral gavage with MCE 100, 200, and 400 mg/kg/d for 8 weeks. The metformin group was administrated by gavage with metformin 50 mg/kg/d for 8 weeks. At the end of the experiment, the rats were sacrificed to collect blood in the tip of the heart under the condition of anaesthesia using 120 mg/kg pentobarbital sodium after the body weight was measured. At the same time, a part of liver and skeletal muscle tissue were dissected and frozen immediately at −80 °C.

### Measurement of fasting serum parameters

The fasting serum glucose was measured by the automatic biochemical analyzer (7600-20 type, Hitachi High-Technologies, Tokyo, Japan). The levels of fasting serum insulin, TNF-α, and IL-6 were measured by ELISA using a commercial assay kit according to instructions from the manufacturer (rat insulin, TNF-α, and IL-6 ELISA kit, R&D Corporation, Minneapolis, MN). Homeostasis model of assessment-insulin resistance (HOMA-IR) was calculated by the following formula: fasting serum glucose (mmol/L) × fasting serum insulin (mIU/L)/22.5.

### Hepatocyte glycogen analysis

Small pieces of liver tissue were fixed with neutral buffered formalin (200 g/kg) solution and embedded in paraffin for periodic Acid-Schiff staining (Nanjing Jiancheng Corporation, Nanjing, China). Under microscope (Olympus, Tokyo, Japan, BH53), the images were photographed at 10 (ocular) × 40 (object lens) magnification, and the average adipocyte area was computed with Image-Pro Plus 6.0 software (NIH Image J system, Bethesda, MD).

### Reverse transcription-PCR analysis

The total RNA of liver from rats was isolated with a Trizol Reagent according to the instruction from the manufacturer. The total RNA was subjected to reverse transcription into cDNA in 100 µL reaction mixture. Polymerase chain reaction (PCR) was performed in a final 20 µL mixture using primer sequences under PCR conditions as listed in Table 1. The products were run on 2% agarose gels and stained with ethidium bromide. The relative density of the band was evaluated using Gene Genius Match systems software (BIO-RAD, Hercules, CA). The relative quantification results of gene expression were normalized on GAPDH transcript levels. Results were expressed as the mean of three independent experiments performed in triplicate.

**Table 1.** Primers used in this study.

Gene	Forward primer and reverse primer	Annealing temperature (°C)	PCR product (bp)	Accession number
GAPDH	F:5′-TCTCCGCCCCTTCCGCTGAT-3′ R:5′-CCACAGCCTTGGCAGCACCA-3′	57	291	NM_017008
Akt-2	F:5′-ATGGTAGCCAACAGTCTGAAGC-3′ R:5′-TTGCCGAGGAGTTTGAGATAAT-3′	55	167	NM_017093
PTP-1B	F:5′-TGCACAGCATGAGCAGTATG-3′ R:5′-TGTGCCTTTTGTTCCTCCTC-3′	55	133	NM_012637
SOCS-3	F:5′-ACCAGCGCCACTTCTTCACA-3′ R:5′-GTGGAGCATCATACTGGTCC-3′	55	450	NM_053565
JNK	F:5′-AGTGTAGAGTGGATGCATGA-3′ R:5′-ATGTGCTTCCTGTGGTTTAC-3′	55	182	NM_053829

### Western blot analysis

Skeletal muscle and liver samples were powdered with ultrasonic cell crush. Each homogenate (10 µL) was mixed with an equal amount of standard SDS sample loading buffer containing 0.25% bromophenol blue, and subjected to SDS-PAGE electrophoresis. After that, protein was transferred onto PVDF membrane by electroblotting. The membranes were then treated sequentially with blocking solution, and then with anti-GLUT-4, anti-Akt-2, anti-PTP-1B, anti-SOCS-3, or anti-JNK antibody (RD Inc, Minneapolis, MN). After washing, membranes were incubated with HRP-labelled secondary antibody (DAKO, Carpinteria, CA). Finally, electrochemiluminescence (ECL) was used to visualize the bands.

### Statistical analysis

The results are presented as mean ± standard deviation. One-way analysis of variance (ANOVA) was used to determine the overall significant difference, and Fisher’s least significant difference (LSD) procedure was used to determine the significant differences between two groups. SPSS 17.0 statistical software (SPSS, Chicago, IL) was used for statistical analysis. *p* < 0.05 was considered as statistically significant.

## Results

### Body weight

**Figure 1.**
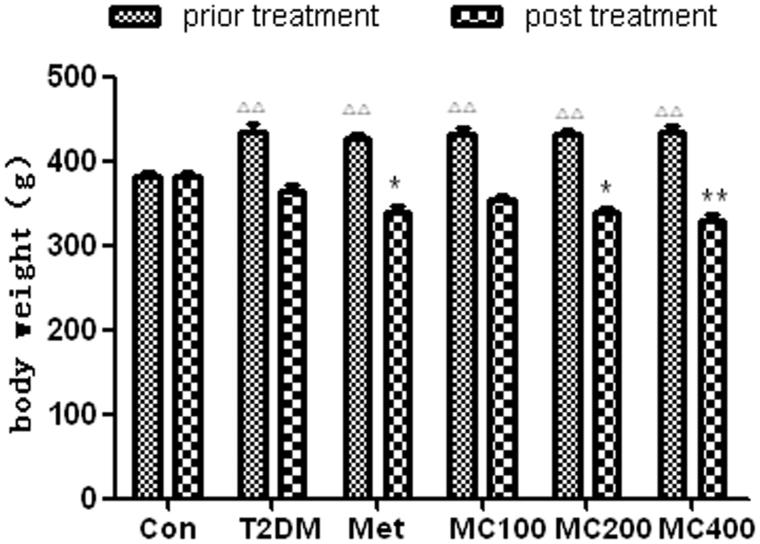
The change of body weight before and after treatment. T2DM: type 2 diabetes mellitus; Met: Metformin; MC100: MCE 100 mg; MC200: MCE200 mg; MC400: MCE400 mg. *p < 0.05 and **p < 0.01 the T2DM group versus control and treated groups; ΔΔ*p* < 0.01 the pretreatment control group versus the pretreatment T2DM groups.

As shown in Figure 1, before treatment, the body weight increased obviously in the model diabetic rats, compared with that in the control rats (*p* < 0.01). Compared with prior treatment, the body weight decreased in all diabetic rats after treatment, however, the body weight decreased obviously in Met (*p* < 0.05) and in MCE 200 mg (*p* < 0.05) and 400 mg (*p* < 0.01) groups, compared with that in T2DM model groups without treatment.

### Serum glucose, insulin, and HOMA-IR levels

**Figure 2.**
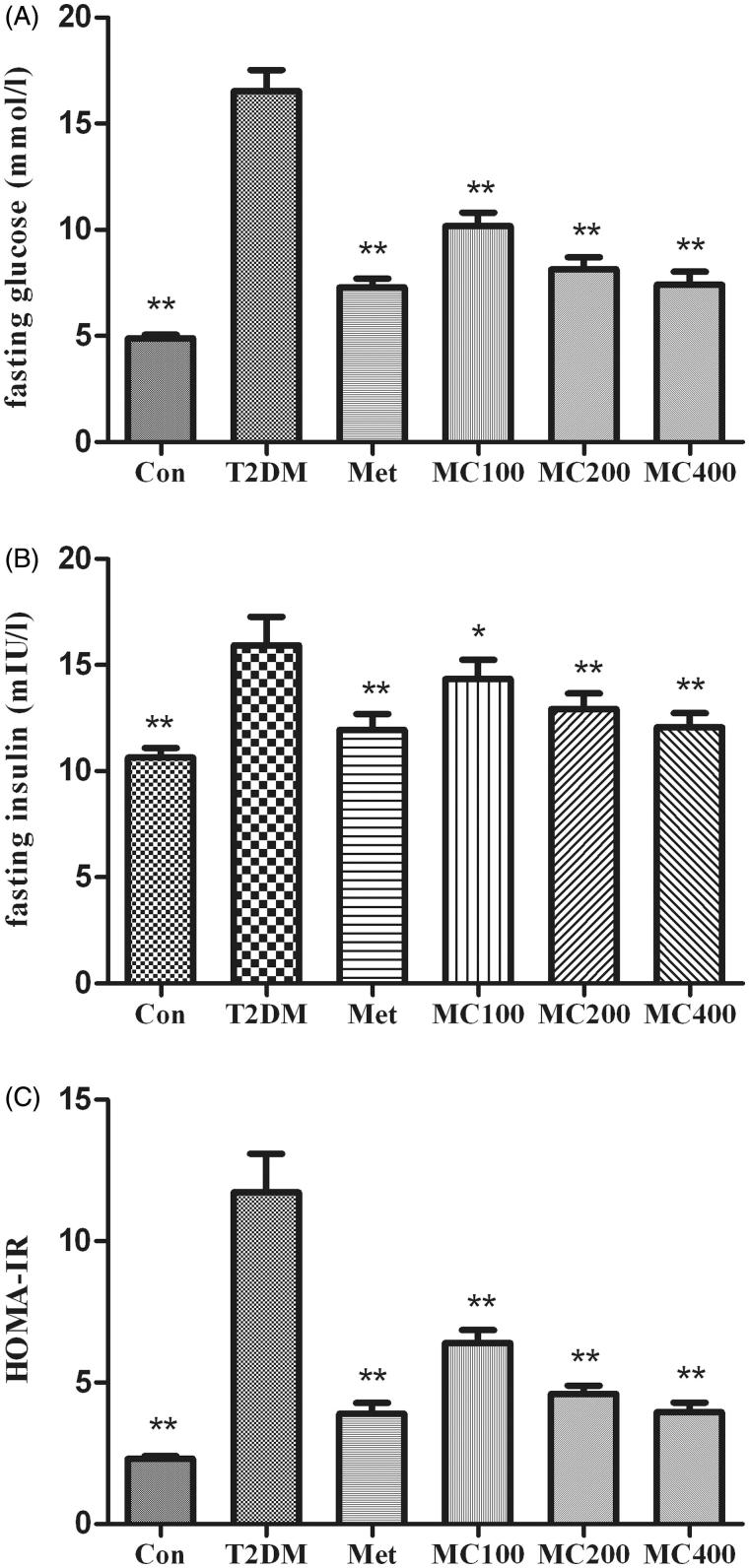
The change of fasting serum glucose, insulin, and HOMA-IR. After 8 weeks treatment, following an overnight fasting, rats were sacrificed and the serum was assay. (A) fasting serum glucose, (B) fasting serum insulin, (C) HOMA-IR; Con: control; T2DM: Type 2 diabetes mellitus; Met: Metformin; MC100: MCE 100 mg; MC200: MCE 200 mg; MC400: MCE400 mg. *p < 0.05 and **p < 0.01 the T2DM group versus control and treated groups.

At the beginning and the end of the experiments, fasting serum glucose, insulin, and HOMA-IR are measured as shown in Figure 2. An obvious decrease in fasting serum glucose was observed in MCE group rats compared with T2DM rats (*p* < 0.01). Fasting serum insulin levels were elevated in the T2DM group compared with that from the control group; however, after 8 weeks treatment, MCE was expected to decrease these values in the treated diabetic group (*p* < 0.05). The result of HOMA-IR was as same as the fasting serum glucose.

### Liver glycogen content

**Figure 3.**
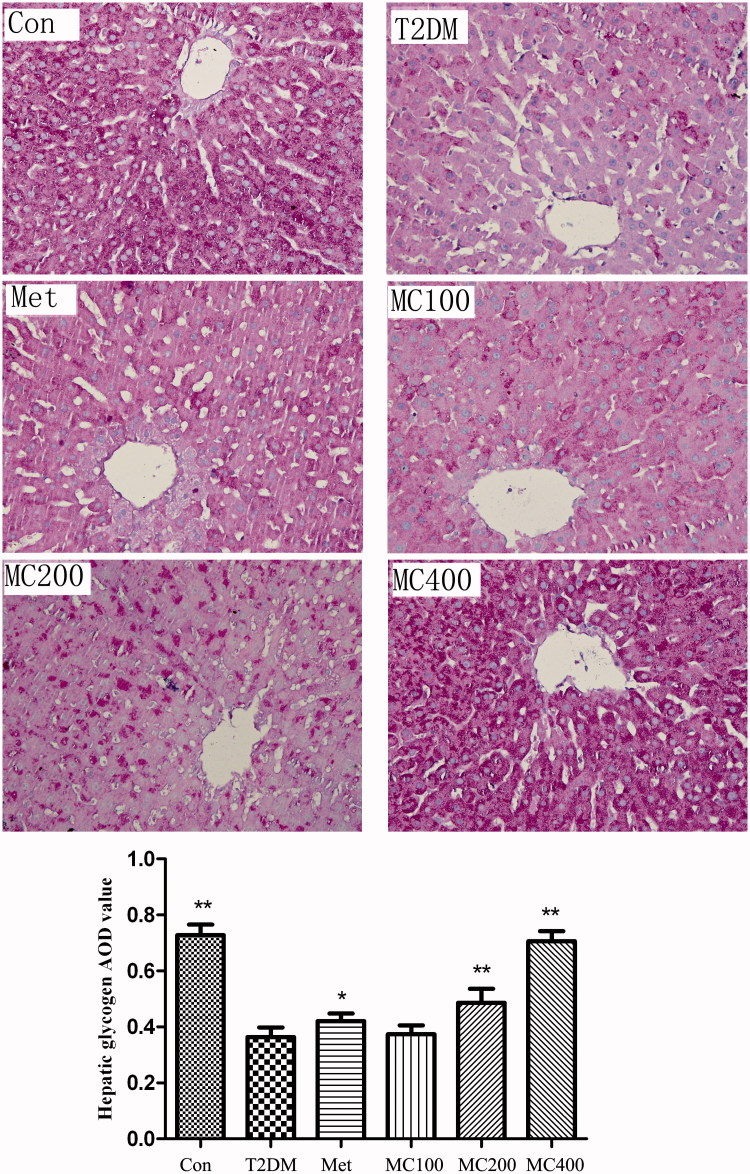
The change of liver glycogen. The result of liver glycogen PAS dyeing (×400), Con: control; T2DM: Type 2 diabetes mellitus; Met: Metformin; MC100: MCE 100 mg; MC200: MCE 200 mg; MC400: MCE 400 mg. *p < 0.05 and **p < 0.01 the T2DM group versus control and treated groups.

The liver glycogen presents fuchsia and the cell nucleus present blue by PAS dyeing (Figure 3). The effects of MCE on the liver glycogen were seen to the figure. The fuchsia liver glycogen granule distributes equality in the normal control group. Through the Image-Pro Plus software analysis (NIH Image J system, Bethesda, MD), the liver glycogen granule’s AOD value in the T2DM group was less than that of the CON group (*p* < 0.01), the liver glycogen granule’s AOD value in 200 and 400 mg (*p* < 0.01) MCE group is more than that of the T2DM group, the same situation exists in the Met group; however, the value (*p* < 0.05) is lower than that of the 200 and 400 mg MCE group.

### Serum inflammatory factor

**Figure 4.**
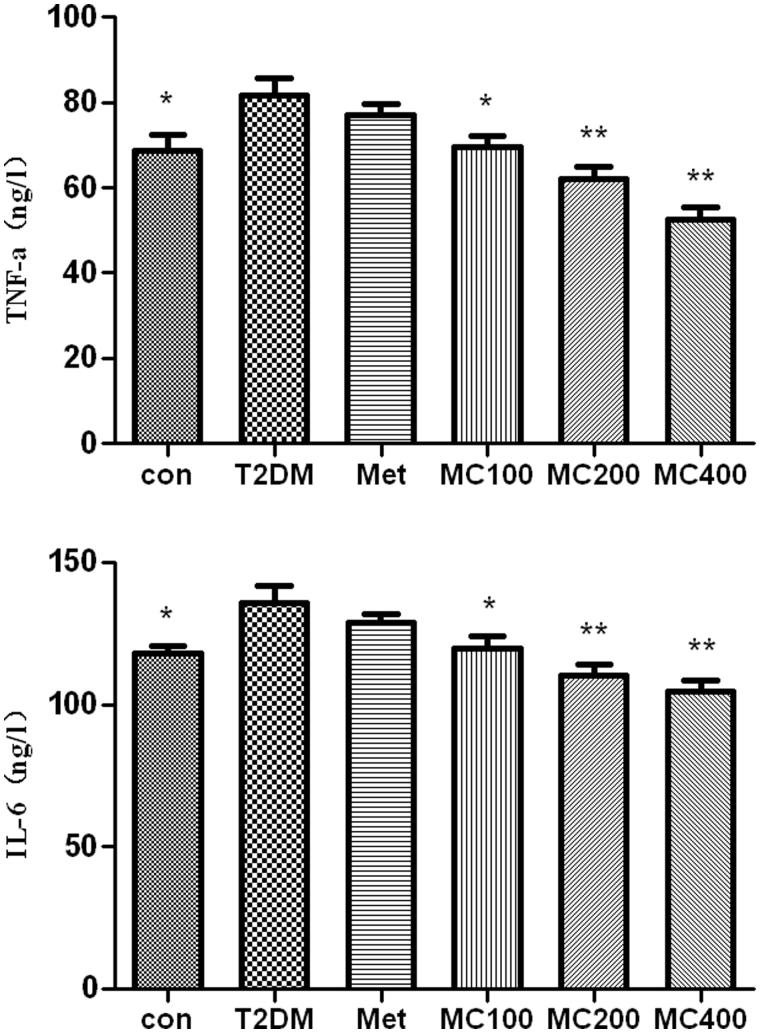
The change of serum inflammatory factors. Con: control; T2DM: type 2 diabetes mellitus; Met: Metformin; MC100: MCE 100 mg; MC200: MCE 200 mg; MC400: MCE 400 mg. *p < 0.05 and **p < 0.01 the T2DM group versus control and treated groups.

As shown in Figure 4, the serum levels of TNF-α and IL-6 were higher in the T2DM than the CON groups (*p* < 0.01). Their levels decreased obviously in different MCE treatment groups, compared with that in the T2DM group (*p* < 0.05 or *p* < 0.01). There was obviously significant difference among each MCE groups (*p* < 0.01) by using LSD statistic method.

### Reverse transcription PCR analysis

**Figure 5.**
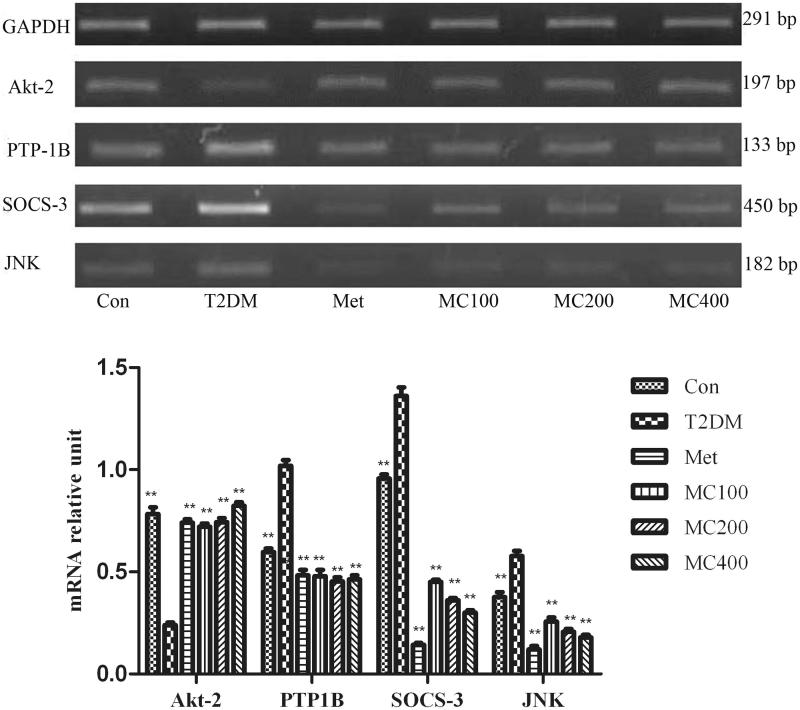
The mRNA expression level of insulin signal transduction pathway relative factors. After 8 weeks treatment, following an overnight fasting, rats were sacrificed and mRNA expression analysis of hepatic insulin regulating factors. Con: control; T2DM: Type 2 diabetes mellitus; Met: Metformin; MC100: MCE 100 mg, MC200: MCE 200 mg; MC400: MCE400 mg. Each value represents the mean ± SE, *n* = 3 rats. **p < 0.01, the T2DM group versus control and treated groups.

Insulin signal pathway factor mRNA expression results are shown in Figure 5. In the untreated diabetic group, *Akt-2* gene expression levels were significantly decreased compared with that from the control group (*p* < 0.01). After treatment of MCE, *Akt-2* gene expression levels were significantly increased compared with that of the untreated diabetic group (*p* < 0.01). The mRNA expression of PTP-1B, SOCS-3, and JNK was increased obviously in the untreated diabetic group, compared with that from the normal group, and the expression level decreased obviously in all treatment groups, compared with that from the untreated diabetic group (*p* < 0.01). The mRNA expression levels of SOCS-3 and JNK were reduced with the increasing dosage of MCE (*p* < 0.01).

### Western blot analysis

**Figure 6.**
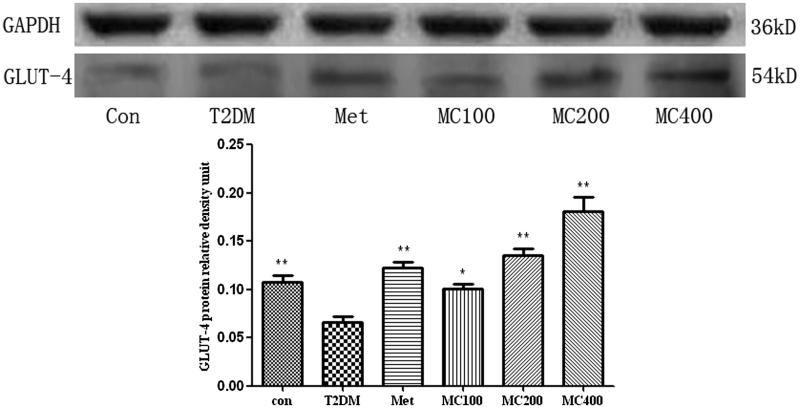
The protein level of GLUT-4 in skeletal muscle. Data are representative images for GLUT-4 level after 8 weeks treatment. The scanned bar graph shows the GLUT-4’ statistical change. Con: control; T2DM: Type 2 diabetes mellitus; Met: Metformin; MC100: MCE 100 mg; MC200: MCE 200 mg; MC400: MCE 400 mg. *p < 0.05 and **p < 0.01 the T2DM group versus control and treated groups.

GLUT4 and the key factors of insulin signal transduction pathway protein levels were detected by western blot in skeletal muscle and liver tissues. The ratio to GAPDH was used as the relative protein level of each protein. We observed an obvious decrease in the GLUT-4 protein level in the T2DM group, compared with that from the control group (*p* < 0.01). After 8 weeks treatment, the GLUT-4 protein level in different MCE groups was higher than that that from the T2DM group (*p* < 0.01, Figure 6).

**Figure 7.**
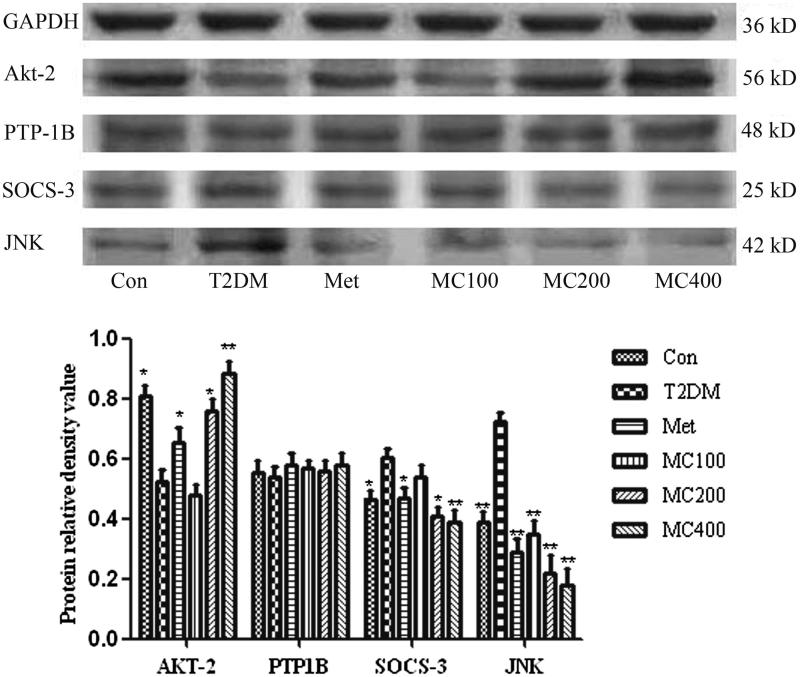
The protein level of insulin signal transduction pathway relative factors. Data are representative images for insulin regulating factors after 8 weeks treatment. The scanned bar graph shows the insulin regulating factors’ statistical change. Con: control; T2DM: type 2 diabetes mellitus; Met: Metformin; MC100: MCE 100 mg; MC200: MCE200 mg; MC400: MCE400 mg. Each value represents the mean ± SE, *n* = 3 rats. *p < 0.05 and **p < 0.01 the T2DM group versus control and treated groups.

The p-Akt (Ser473) protein level was lower in the T2DM group than that from the normal group, but the protein level was increased obviously in MCE 200 and 400 mg groups, compared with that from the T2DM group (*p* < 0.05 and *p* < 0.01). The protein level of SOCS-3 and JNK was obviously higher in the T2DM group than that from the normal group (*p* < 0.05 and *p* < 0.01), but it was decreased obviously in all treatment groups compared with that from the T2DM group (*p* < 0.05 and *p* < 0.01), and the protein level decreased with the increasing dosage of MCE (Figure 7). The protein level of PTP-1B was increased obviously in the T2DM model group, compared with that from the normal group (*p* < 0.01).

## Discussion

Obesity is a common inducement in IR. In our research, MCE has the effect of weight loss of diabetic rats, that is to say, MCE treatment can reduce the IR’s possibility of diabetic rats. Our findings confirmed that MCE could reduce the fasting serum glucose and insulin level in T2DM rats through 8 week’s treatment. The hypoglycaemic effect of 400 mg MCE group was similar to metformin as reported previously (Miura et al. 2001). Because MCE treatment lowered the fasting serum insulin level, we speculate that the hypoglycaemic effect of MCE is to ameliorate insulin resistance rather than stimulate the pancreatic β-cell to secret insulin. Nowadays, homeostasis model assessment insulin resistance (HOMA-IR) has become the common index to evaluate the insulin sensibility, insulin resistance level, and the function of pancreatic β-cell (Fujino et al. 2013). In our research, the HOMA-IR in T2DM rats was higher than the control group and decreased obviously after MCE treatment, indicating that MCE might obviously ameliorate insulin resistance.

After the meal, hepatic glycogen synthesis and storage is increased. When blood glucose was decreased because of too much utilization, hepatic glycogen is decomposed and released to increase the blood glucose, which is regulated by insulin, glucagon, adrenaline, etc. (Roach et al. 2012). In our study, the liver glycogen level in 200 and 400 mg MCE group is more than that of the T2DM group. This shows that MCE has the effect of increasing glycogen contents. Hazarika et al. (2012) found that MCE could prevent the phosphorylation of glycogen synthase kinase-3 (GSK-3) which might inhibit the activity of glycogen synthase and promote the synthesis of glycogen. Because the expression level of GLUT-4 can reflect the utilization degree of glucose, the promoting effects of MCE treatment on the GLUT4 expression demonstrated that MCE could decrease blood glucose by up-regulating GLUT expression, in line with previous reports (Chun et al. 2009; Kumar et al. 2009). The higher expression of GLUT-4 is the key factor for glucose utilization in peripheral tissue via the adenosine monophosphate-activated protein kinase (AMPK) pathway. MCE exerted anti-diabetic properties in HF-fed mice, also evidenced by a high AMPK phosphorylation and a low hepatic glucose production (Ojuka et al. 2002).

Reportedly, there are three Akt subtypes in mammals and Akt-2 deficiency showed the abnormal blood insulin and glucose. Akt activation caused glycogenesis, restraining glycogenolysis, restraining gluconeogenesis, and enhancing glucose oxidization and utilization (Sharma et al. 2015). The phosphorylation of Akt-2 at serine 473 is responsible for its activation (Tsuchiya et al. 2014) and employed to indicate the MCE effect on insulin signal transduction pathway. Here, the mRNA and protein expression levels of Akt-2 were increased obviously in MCE groups, and increased with the dosage of MCE, suggesting that MCE might improve the insulin signal transduction through up-regulating the Akt-2 expression.

There are some important negatively regulatory proteins in the course of Akt’s activation, such as PTP-1B, SOCS-3, and JNK, whose down-regulation can weaken the inhibition effect of insulin and ameliorate IR. Some inflammatory factors (e.g. TNF-α and IL-6) can increase the mRNA expression of PTP-1B through NF-κB pathway (Yoshihiro et al. 2012), activate SOCS-3 (Shi et al. 2004), and JNK pathway (Choi et al. 2012). They finally influence the phosphorylation of insulin-sensitive cells, prevent the insulin signal transduction pathway, reduce the expression of GLUT-4, and then induce IR (Hashimoto et al. 2010; Kalupahana et al. 2012). In our studies, the treatment of MCE can decrease the serum TNF-α and IL-6 levels obviously, which gives another explanation about the inhibitory effects of MCE on IR.

PTP-1B is a member of protein tyrosine phosphatase family and its inhibition is the potential therapeutic way for obesity, insulin resistance, and T2DM (Koren and Fantus 2007). In our research, the mRNA expression amount of PTP-1B in MCE groups was lower than in the T2DM group. However, the change of PTP-1B mRNA level was not related with the dosage of MCE and was inconsistent with the change of protein level, demonstrating that the effect of MCE on ameliorating IR was independent of PTP-1B’s expression. This may be associated with post-transcriptional control. SOCS family is a group of negative regulatory protein of cytokines through the central SH2 structural domain and conservative c-terminus box (Wormald and Hilton 2004). SOCS-3 can regulate the insulin sensibility through inhibiting tyrosine phosphorylation of insulin receptor substrate-1 and control insulin action and glucose homoeostasis (Galic et al. 2014). The treatment of MCE decreased the mRNA and protein expression level of SOCS-3 with the increasing dosage of MCE. JNK is one of the members of mitogen-activated protein kinase (MAPK) family and widely participates in cell differentiation, apoptosis, immunoreactions, and IR. JNK overexpression can result in IR through the inhibition of insulin receptor substrate (IRS1): phosphatidylinositol 3-kinases (PI3K) signal transduction (Sharfi and Eldar-Finkelman 2008). Here, the treatment of MCE down-regulated the mRNA and protein expression level of JNK with the increasing dosage of MCE. MCE can inhibit phosphorylation of JNK and nuclear translocation of NF-κB, which contribute to the improvements of insulin signalling and inflammation (Soo et al. 2015). These results showed that MCE ameliorated IR by down-regulating SOCS-3 and JNK mRNA and protein expression.

In conclusion, the hypoglycaemic and ameliorative effects of MCE on IR might be closely linked to the increases in hepatic glycogen, peripheral tissue’s GLUT-4 expression, and higher insulin sensitivity by down-regulating the expression of SOCS-3 and JNK. This study can afford to the theoretical and experimental supports to exploit a new type of drug in T2DM personalized treatment.
